# Genome editing for sustainable agriculture in Peru: advances, potential applications and regulation

**DOI:** 10.3389/fgeed.2025.1611040

**Published:** 2025-06-30

**Authors:** Marilu Mestanza, Angel David Hernández-Amasifuen, Alexandra Jherina Pineda-Lázaro, Dennis Eriksson, Juan Carlos Guerrero-Abad

**Affiliations:** ^1^ Escuela de Posgrado, Programa Doctoral en Ciencias para el Desarrollo Sustentable, Facultad de Ingeniería Zootecnista, Biotecnología, Agronegocios y Ciencia de Datos, Universidad Nacional Toribio Rodríguez de Mendoza de Amazonas, Chachapoyas, Peru; ^2^ Instituto de Investigación, Innovación y Desarrollo para el Sector Agrario y Agroindustrial (IIDAA), Facultad de Ingeniería y Ciencias Agrarias, Universidad Nacional Toribio Rodríguez de Mendoza de Amazonas, Chachapoyas, Peru; ^3^ Department of Plant Breeding, Swedish University of Agricultural Sciences, Alnarp, Sweden

**Keywords:** CRISPR/Cas, Ribonucleoproteins (RNP), tRNA-like sequence (TLS), gene editing, “transgene-free” tool, Peruvian moratorium, sustainable Peruvian agriculture

## Abstract

Peruvian agriculture is characterize by crops such as potato, maize, rice, asparagus, mango, banana, avocado, cassava, onion, oil palm, chili, papikra, blueberry, coffee, cacao, grapes, quinoa, olive, citrus and others. All of them have challenges in production in their specific agroecosystems under stress due to pests, diseases, salinity, drought, cold among others. Gene editing through CRISPR/Cas is a key tool for addressing critical challenges in agriculture by improving resilience to biotic and abiotic stress, increasing yield and enhancing the nutritional value of the crops. This approach allows precise mutation on site-specific gene at the DNA level, obtaining desirable traits when its function is altered. The CRISPR/Cas system could be used as a transgene-free genome editing tool when the ribonucleoprotein (RNP) acts as a carrier to delivered the CRISPR/Cas components into the plant cell protoplasts, or when the tRNA-like sequence (TLS) motifs are fused to single-guide RNA (sgRNA) and Cas mRNA sequence and expressed in transgenic plants rootstock to produce “mobile” CRISPR/Cas components to upper tissue (scion). Those innovations could be a potential approach to strengthen the Peruvian agriculture, food security and gricultural economy, especially in the tropical, Andean and coastal regions. This review article examines the advances and strategies of gene editing, focusing on transgene-free methodologies that could be adopted for research, development and use, and also identifies potential applications in key crops for Peru and analyzes their impact in the productivity and reduction of agrochemicals dependence. Finally, this review highlights the need to establish regulatory policies that strengthen the use of biotechnological precise innovations, ensuring the conservation and valorization of agrobiodiversity for the benefit of Peruvian farmers.

## 1 Introduction

The agricultural system presents various challenges during the production cycle of different crops (Nicholson et al., 2021; Rodriguez et al., 2024). Given that, external factors such as biotic and abiotic stress, intensive resource exploitation (p.e: water and soil) and extreme climatic events (Herring, 2004; Yuan et al., 2024), turns unfavorable conditions for food production and availability of essential resources in the crops, contributing significantly to global food insecurity. In this context, gene editing mediated by CRISPR/Cas system is a promising tool to develop resilient crops, that allows precise mutation on site-specific gene at the DNA level to display new traits than can be incorporated and used in the plant breeding programs (Ceasar and Kavas, 2024; Ndudzo et al., 2024).

To get a loss of function of a site-specific gene, Zinc Finger Nucleases (ZFNs) (Davies et al., 2017) and Transcription Activator-Like Effector Nucleases (TALENs) (Sprink et al., 2015) have been used, but over the time have presented some limitations (Gaj et al., 2013) in their accuracy and cost. However, CRISPR/Cas system has emerged as a transformative breakthrough (Cardi et al., 2023) given its simplicity, efficiency and ease to use for many scientists that have adapted it as a reliable tool for genome editing on different organisms, including plants (Gan and Ling, 2022). Also, could be considered a “transgene-free” tool (i.e., without stable insertion of any transgenes) when the ribonucleoprotein (RNP) is used as a carrier of the CRISPR/Cas components into the protoplasts (Tiwari et al., 2022) or when tRNA-like sequence (TLS) motifs are fused to single-guide RNA (sgRNA) and Cas mRNA sequence and expressing in transgenic plants rootstock, enabling systemic mobility of CRISPR/Cas system components through to others tissues of the scion (Yang et al., 2023). In addition to RNP-based methods, other transgene-free strategies have been developed, including the use of *in vitro* transcripts (IVTs) (Liang et al., 2018) and viral replicons (Shen et al., 2024). These molecules can be introduced not only into protoplasts but also directly into intact plant tissues through delivery techniques such as biolistics. Moreover, the co-editing strategy has recently gained attention as an efficient and promising alternative for transgene-free genome editing (Jia et al., 2024). All these strategies rely on the same principle: sgRNA designed based on the site-specific target gene leads a Cas protein to make a precise cut in the gene, enabling a modification without the need for stable transgene integration (Workman et al., 2021).

The Clustered Regularly Interspaced Short Palindromic Repeats, also called CRISPR discovered in bacteria, mostly employs the Cas9 protein; however additional Cas variants exhibiting distinct properties, such as Cas12 (previously Cpf1), Cas13 and Cas14, which makes gene editing even more versatile (Hillary and Ceasar, 2023; Movahedi et al., 2023). In addition, base editing technology is a promising alternative because it allows even higher precision in the design of exact base edits without generating double-strand breaks (Azameti and Dauda, 2021). Therefore, these genome editing tools are considered as next-generation strategies for plant breeders to modify specific genes of the whole genome to generate resilient crops to biotic and abiotic stresses and, therefore, reduce herbicide and insecticide products in the production system.

Peru is a megadiverse country that offers a wide diversity of crops (Pearsall, 2008; MIDAGRI, 2025b) such as potato, maize, rice, asparagus, mango, banana, avocado, blueberry, coffee, cacao, grapes, quinoa, olive, citrus among others (Porro et al., 2015; Schwarz et al., 2019). Its economy depends in part on the agriculture and livestock sector, which accounts for approximately 6% of the national Gross Domestic Product (GDP), with crop production representing more than 60% of this value (Banco Central de Reserva del Perú, 2024). According the latest 2017 Census, more than 20% of the population resides in rural areas (INEI, 2017), where 78% is dependent on agriculture for their livelihood (Cabrera-Cevallos and De la O Campos, 2023). Likewise, agro-export companies have maintained a sustained growth, reaching a value of US$ 1,314 million in January of this year, which represents an increase of 23.3% compared to the same month of 2024. Among the main exported products, blueberries are the second most exported agroindustrial product, after grapes, with a value of US$ 128 million (ADEX, 2025).

Biotechnological innovations could play a key role in strengthening the country’s food security and agricultural economy (Potter et al., 2023). In the Peruvian context, the cultivation of genetically engineered (GE) crops meeting the legal definition of living modified organisms (LMOs) is currently limited by the moratorium (Law no. 29811) approved by the Congress in 2011 and subsequently extended in 2021 for another 15 years, through Law no. 31111, however there are three exceptions: 1) laboratory research; 2) use in pharmaceuticals and veterinary products; and 3) use in food, animal feed, and in food processing. It means that it is not possible planting GE crops (also called as transgenic crops) in the agricultural fields around the Peruvian territory. According The Cartagena Protocol on Biosafety (CPB), an LMO is defined as “*any living organism that possesses a novel combination of genetic material obtained using modern biotechnology*” (Secretariat of the Convention on Biological Diversity, 2000). Based on this, the CRISPR/Cas system could be considered as a transgene-free tool when the RNPs technology is employed into protoplasts, avoiding the integration of exogenous DNA into the plant genome (Kim et al., 2024) or when the CRISPR/Cas sequences system is fused to the TLS motifs, thus allowing its transport from the rootstock to multiple scion tissues, this approach enables the generation of mutated seeds without the need to incorporate transgenic sequences (Hu and Gao, 2023; Yang et al., 2023). Both strategies could provide a pathway solution for biotechnological restrictions for cultivation of genetically engineered (GE) organisms.

This review article examines the gene editing advances and strategies, focusing on transgene-free methodologies that could be adopted in the Peruvian agriculture for research, development and access. And also emphasizes the potential applications in key crops for Peru and analyzes their impact on productivity and reduction of agrochemicals dependence. Finally, the review focus on the need for evidence-based regulatory policies that strengthen the use of biotechnological precise innovations in the Peruvian agriculture.

## 2 CRISPR/Cas advances and applications in plants

**FIGURE 1:**
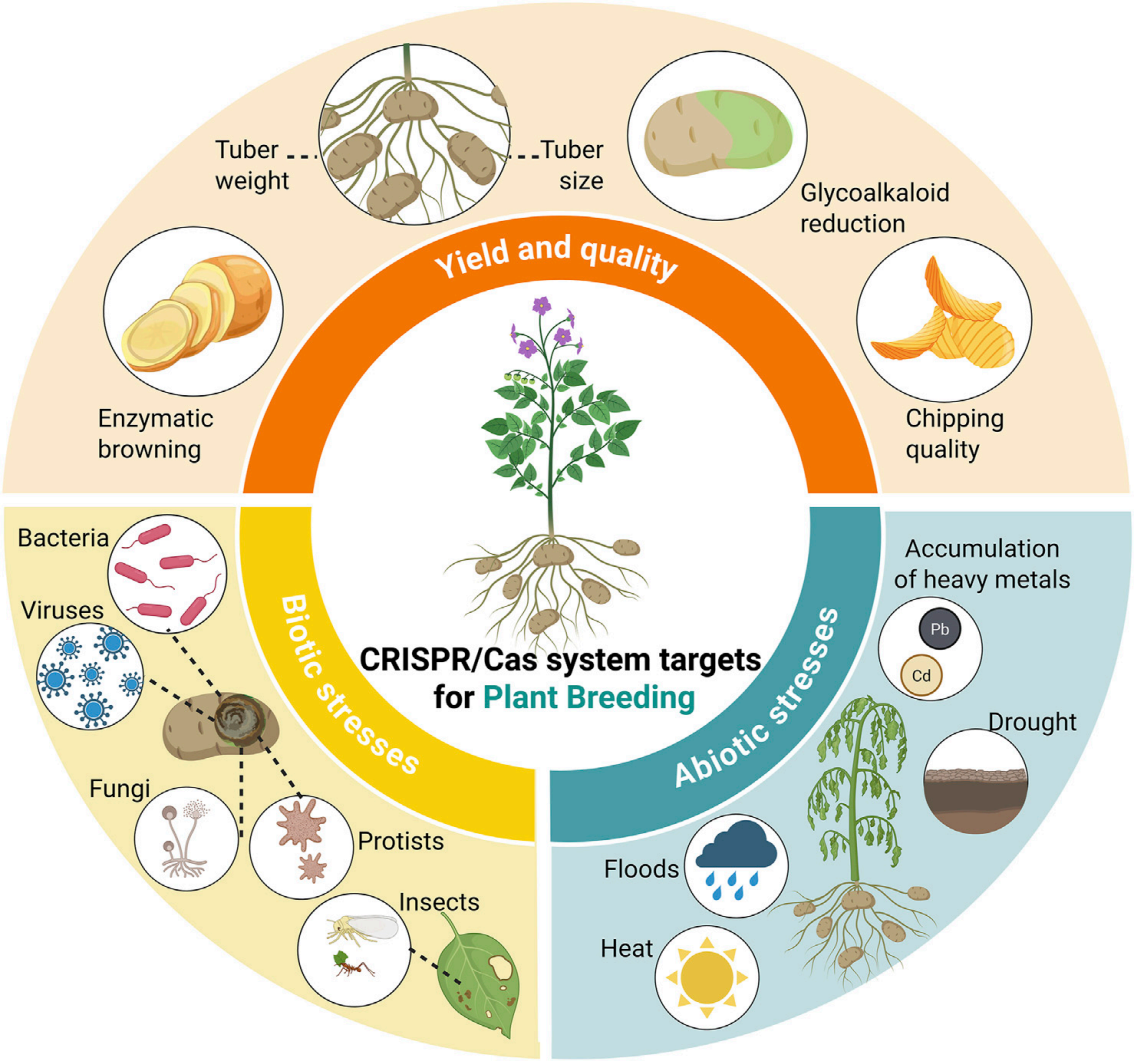
Potential targets of plant gene editing using CRISPR/Cas system for potato improvement. Potato and other crops can be also improved by addressing any possible regulator of yield, quality, and biotic or abiotic stress tolerance.

CRISPR/Cas technology has overcome numerous limitations associated with traditional breeding techniques (Muha-Ud-Din et al., 2024). Major achievements include increased resistance to pathogens, tolerance to unfavorable environmental conditions, and improved nutritional quality of crops, which are essential for enhancing food security and adapting agriculture to climate change (González et al., 2020; Shinwari et al., 2020) (Figure 1).

**FIGURE 2:**
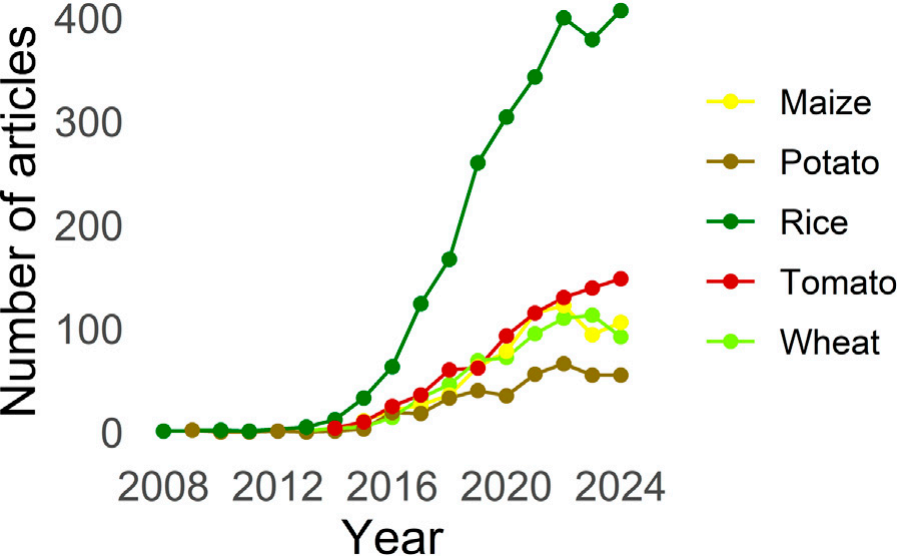
Bibliometric analysis of CRISPR/Cas application in different crops for specific traits.

Precise edits using the CRISPR/Cas system have been achieved to improve disease resistance and increase yield in cereal, fruit, vegetable and tuber crops. Much of the research in recent years has focused on the three staple food crops: rice, wheat, and maize (Xu et al., 2024; Li et al., 2022; Liu et al., 2021), reflecting the strategic importance in sustainable food production. A bibliographic analysis based on Web of Science data confirm the importance of CRISPR/Cas system in the research field (Figure 2). Rice stands out as the crop with the highest number of scientific publications (2,501 articles), showing an increasing trend in gene editing studies over the years. It is followed by tomato (822 articles), maize (675 articles), wheat (654 articles) and potato (384 articles), reflecting the scientific community’s interest in optimizing these crops using biotechnological tools.

Rice (*Oryza sativa* L.), a staple food in the diet of more than half the world’s population, plays a vital role in food security, especially in Asia, where it is often the primary source of nutrients. In Peru, rice is also a key component of the national diet, with a *per capita* consumption of approximately 54 kg per year, the highest in Latin America (INEI, 2024). Its production is concentrated in the departments of San Martin, Piura, Lambayeque, La Libertad and Amazonas, which together accounted for 72.36% of the national rice production of 2024 (MIDARGI, 2025a). Despite its importance, rice production faces significant challenges against several plant pathogens, including *Meloidogyne graminicola*, a root-knot nematode (RKN) responsible for yield losses between 17% and 32% (Kyndt et al., 2014; Mantelin et al., 2017). Most rice cultivars are highly susceptible to this plant-parasitic nematode, and changes in agricultural practices have exacerbated its prevalence (Mantelin et al., 2017). Numerous investigations on gene editing have been carried out in response to this challenge and the search for improvements in cultivation (Table 1).

**TABLE 1:** CRISPR/Cas applications in cereal crop improvement*.

Crop	Edited gene	Improved trait	References
*Hordeum vulgare* (barley)	*GST, IPI, PDI, CRT, HSP70, HSP26, HSP16.9*	Enhanced recombinant protein yield and distribution	Panting et al. (2021)
	*PTST1, Gbss1a*	Increased amylose content and improved grain viability	Zhong et al. (2019)
*Oryza sativa* (rice)	*LKR/SDH*	Increased lysine content in grains without affecting agronomic traits	Rastogi et al. (2025)
	*OsCKX1–OsCKX11*	Increased panicle size, grain number, and altered seed morphology and starch composition	Zheng et al. (2023)
	*OsHPP04*	Improved resistance to root-knot nematode without yield penalties	Huang et al. (2023)
	*Waxy*	Optimized amylose content for better cooking and eating quality	Huang et al. (2020)
	*CrtI, PSY*	Increased carotenoid content without trade-offs	Dong et al. (2020)
	*OsGAD3*	Increased GABA levels, grain weight, and protein content	Akama et al. (2020)
	*OsBADH2*	Enhanced aroma for better sensory quality and market value	Ashokkumar et al. (2020)
	*OsPIN5b, GS3, OsMYB30*	Improved panicle length, grain size, and cold stress tolerance	Zeng et al. (2020)
	*OsGS3, OsGW2, OsGn1a*	Enhanced grain size, width, weight, and number	Zhou et al. (2019)
	*OsPLDα1*	Reduced phytic acid to improve micronutrient bioavailability	Khan et al. (2019)
*Triticum aestivum* (wheat)	*TaARF12*	Reduced plant height, larger spikes, and increased grain yield	Kong et al. (2023)
	*TaGW7*	Increased grain width and weight; decreased grain length	Wang et al. (2019b)
*Zea mays* (maize)	*CLE*	Increased meristem size and grain yield traits	Liu et al. (2021)
	*Wx1*	Increased amylopectin content and waxy corn yield	Gao et al. (2020a)
	*SH2, GBSS*	Higher sugar and amylopectin levels in sweet and waxy maize	Dong et al. (2019)

A prominent example of increasing resistance to *M. graminicola* was conducted using CRISPR/Cas9 system to edit the susceptibility gene *OsHPP04* in rice. Transgene-free mutants showed enhanced immune responses and retained agronomic traits, highlighting the potential of gene editing for nematode-resistant crops (Huang et al., 2023). Another study focused on improving resistance to blast (*Pyricularia oryzae*) by editing the susceptibility genes *OsDjA2* and *OsERF104*. The edited plants showed no adverse effects on growth, thus demonstrating the safety and efficacy of this technique (Távora et al., 2022). In China, research has also been conducted to increase yield (including increased panicle length and grain size) and improve cold tolerance. This was achieved by simultaneously editing three key genes (*OsPIN5b*, *GS3* and *OsMYB30*) using the CRISPR/Cas9 system (Zeng et al., 2020).

Wheat is the second most important staple food globally, providing more than 20% of the daily calories and protein consumed. It is grown in 89 countries and contributes to the diets of approximately 2.5 billion people (Gohar et al., 2022). However, it faces challenges such as climate change and diseases caused by pathogenic fungi, such as rust and ear blight, which threaten its global production (Junk et al., 2016). In response, gene editing offers innovative solutions to ensure their sustainability and nutritional quality in the future (Elsharawy and Refat, 2023).

Since the pioneering study by Wang et al. (2014), which laid the foundation for gene editing in wheat, significant advances have been achieved through the use of CRISPR/Cas9 system. In that regard, other studies achieved editing of the *TaGW7* gene, showing dose-dependent effects on grain morphology, increasing grain width and weight while reducing its length. Similarly, editing *TaARF12* led to yield improvements of up to 11.1% by reducing plant height and promoting more prominent ears and a higher grain number (Table 1). In turn, Wang et al. (2018) implemented a multiplex editing strategy targeting *TaGW2*, *TaLpx-1*, and *TaMLO*, where knockout of *TaGW2* resulted in a significant increase in seed size and grain weight, with heritable effects observed in subsequent generations.

Maize (*Zea mays* L.) has become the most widely grown and traded crop globally, playing a key role in the production of food, feed and biofuels (Erenstein et al., 2022). However, its yield is affected by drought in 20% of the cultivated area for each year, and numerical simulation reveals that yield loss will increase by 14.10%–33.40% during 2020–2050 (Bi et al., 2023). High temperatures (above 32 °C) have also been reported to affect flowering by decreasing pollen viability, thereby reducing fertilization and grain yield (Kumar et al., 2021). In addition, pests and diseases can cause losses exceeding 3.75 million tons per year (Hampf et al., 2021). Hence, it is essential to develop maize cultivars that are more productive, resistant, and tolerant to biotic and abiotic stresses. Examples of improvements with CRISPR/Cas9 include editing the ARGOS8 promoter, which has increased yield under drought conditions (Shi et al., 2017), Furthermore, modifications in *Stiff1* and *ZmGA20ox3* genes have strengthened stalks and created semi-dwarf plants suitable for high-density plantings (Liu et al., 2023). Other examples of yield improvement and disease tolerances are given in (Tables 1–4).

**TABLE 2:** CRISPR/Cas applications in fruit crop improvement*.

Crop	Edited gene	Improved trait	References
*Citrus maxima* (pomelo)	*CsLOB1*	Bacterial canker resistance in citrus achieved in the T_0_ generation	Jia and Wang (2020)
*Citrus sinensis* (sweet orange)	*CsLOB1*	Enhanced resistance to bacterial canker	Huang et al. (2022)
*Fragaria x ananassa* (strawberry)	*RAP*	Improved fruit coloration via modulation of anthocyanin transport	Gao et al. (2020b)
*Malus sieversii* (red-fleshed apple)	*MdMKK9*	Enhanced anthocyanin accumulation and tolerance to low-nitrogen conditions	Sun et al. (2022)
*Musa acuminata* (diploid banana)	*MaACO1*	Delayed ripening and extended shelf life	Hu et al. (2021)
	*LCYε*	Increased β-carotene content with reduced levels of α-carotene and lutein	Kaur et al. (2020)
*Musa spp*. (triploid banana and plantain)	*eBSV*	Resistance to endogenous BSV activation under stress in B genome germplasm	Tripathi et al. (2019)
*Solanum melongena* (eggplant)	*SmelPPO4, SmelPPO5, SmelPPO6*	Reduced fruit browning through targeted suppression of *PPO* genes	Maioli et al. (2020)
*Vaccinium spp*. (blueberry)	*CENTRORADIALIS (CEN)*	Modulated vegetative growth through functional disruption of CEN gene	Omori et al. (2021)
*Vitis vinifera* (grapevine)	*TMT1, TMT2*	Reduced sugar accumulation in grape berries	Ren et al. (2021)

**TABLE 3:** CRISPR/Cas applications in vegetable crop improvement*.

Crop	Edited gene	Improved trait	References
*Brassica napus* (rapeseed)	*BnSFAR4, BnSFAR5*	Higher seed oil content without loss of plant vigor	Karunarathna et al. (2020)
*Brassica oleracea* (chinese kale)	*BoaCRTISO*	Improved leaf coloration via pigment modulation	Sun et al. (2020)
*Brassica rapa* (chinese cabbage)	*BraFLC2, BraFLC3*	Early flowering without vernalization requirement	Jeong et al. (2019)
*Cucurbita maxima* (pumpkin)	*RBOHD*	Improved salinity tolerance via root ion balance	Huang et al. (2019)
*Solanum lycopersicum* (tomato)	*SlAMS*	Reduced pollen viability for male sterility induction	Bao et al. (2022)
	*SlPelo, SlMlo1*	Dual resistance to tomato yellow leaf curl virus and powdery mildew	Pramanik et al. (2021)
	*MAX1*	Resistance to *Phelipanche aegyptiaca* via strigolactone suppression	Bari et al. (2021)
	*SlHyPRP1*	Salinity tolerance via loss of negative regulatory domains	Tran et al. (2021)
	*SlMAPK3*	Heat stress tolerance via ROS and stress pathway regulation	Yu et al. (2019)
	*ALS1, ALS2, ALS3*	Resistance to chlorsulfuron herbicide	Danilo et al. (2019)
	*SlJAZ2*	Resistance to bacterial speck without loss of defense against necrotrophs	Ortigosa et al. (2019)

**TABLE 4:** CRISPR/Cas applications in tuber crop improvement*.

Crop	Edited gene	Improved trait	References
*Ipomoea batatas* (sweetpotato)	*IbGBSSI, IbSBEII*	Improved starch properties via amylose modification	Wang et al. (2019a)
*Manihot esculenta* (cassava)	*MeSSIII-1*	Increased amylose and resistant starch content in the storage roots	Lu et al. (2025)
	*CYP79D1, CYP79D2*	Reduced cyanide levels for enhanced food safety	Gomez et al. (2023)
	*MeSWEET10a*	Increased tolerance to bacterial blight	Elliott et al. (2023)
	*MeCYP79D1*	Reduced linamarin and cyanide content for enhanced food safety	Juma et al. (2022)
	*nCBP-1, nCBP-2*	Improved tolerance to brown streak disease with reduced root necrosis and virus load	Gomez et al. (2019)
*Solanum tuberosum* (potato)	*InvVac, PPO2*	Improved cold storage and bruising resistance via suppression of cold-induced sweetening and enzymatic browning	Massa et al. (2025)
	*StPM1*	Improved resistance to *Phytophthora infestans*	Bi et al. (2024)
	*Parakletos*	Broad-spectrum resistance to biotic and abiotic stresses with increased field yield	Zahid et al. (2024)
	*StDND1, StCHL1, StDMR6-1*	Enhanced resistance to late blight	Kieu et al. (2021)
	*Coilin*	Increased resistance to potato virus Y and stress tolerance	Makhotenko et al. (2019)
	*StPPO2*	Reduced enzymatic browning in tubers	González et al. (2020)
	*S-RN*	Self-incompatibility breakdown for hybrid breeding	Enciso-Rodriguez et al. (2019)

* Note: Only peer-reviewed scientific studies published between 2019 and 2025 are included.

## 3 Strategies to obtain transgene-free genome editing crops

Over the years, gene editing technology has undergone rapid innovation, significantly expanding its range of applications due to its high efficiency and precision. These attributes, combined with their accessibility and versatility, have bring a major transformation in biotechnological solutions, particularly in agriculture sector where the primary goals are to reduce production costs and enhance the yields of crops with agronomic relevance traits (Mohd-Fadhli and Boon-Chin, 2024; Ndudzo et al., 2024).

**FIGURE 3:**
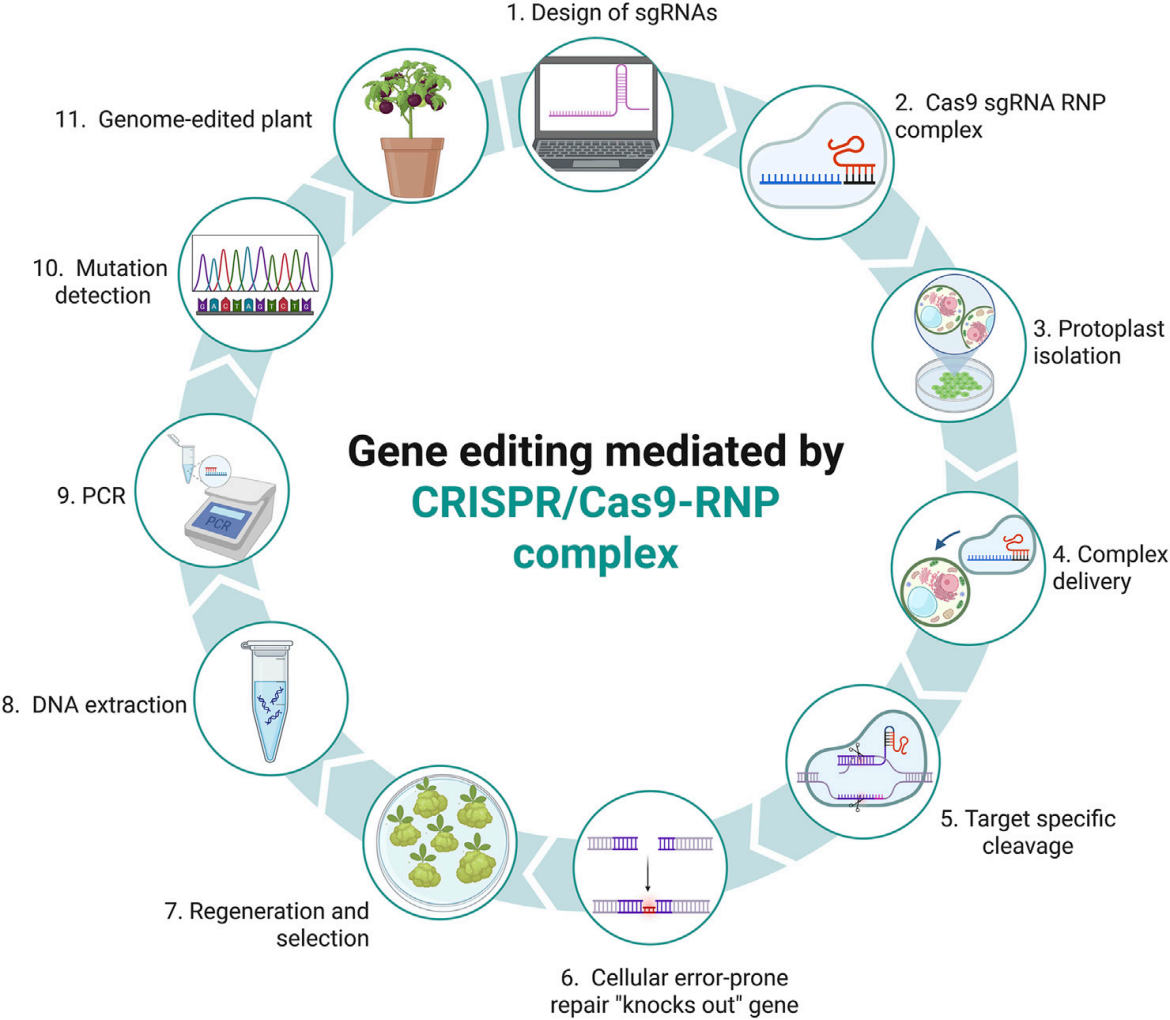
Plant genome editing using a CRISPR/Cas9 ribonucleoprotein (RNP) complex. The process begins with the design of the sgRNA (1), followed by the assembly of the Cas9 protein with single guide RNA (sgRNA) to form the RNP complex (2). Plant protoplasts are isolated (3), and the RNP complex is delivered into the cells (4), where it induces site-specific DNA cleavage (5). The cell’s endogenous repair machinery introduces insertions or deletions at the break site through error-prone non-homologous end joining (NHEJ), leading to gene knockout (6). Regeneration and selection of edited cells are performed (7), followed by DNA extraction (8), PCR amplification (9), and mutation detection (10). Successfully edited plants are then obtained and analyzed (11).

The emergence of the CRISPR/Cas system has provided a more accessible, versatile and efficient alternative for gene editing in plants (Kaul et al., 2025). This system has facilitated the development of transgene-free technologies, such as the direct delivery of ribonucleoprotein (RNP) complexes (Zhang et al., 2021) and the use of TLS motifs (Yang et al., 2023), which are emerging as promising strategies adapted to crops with different *in vitro* transformation and regeneration capabilities. RNPs with CRISPR/Cas complex cleave DNA more rapidly than other delivery methods, and reach a maximum mutation frequency rather soon after transfection, normally within 24 h (Bloomer et al., 2022). Additionally, when delivered as ribonucleoprotein (RNP) complexes, Cas9 degrades more rapidly compared to other delivery methods, thereby reducing the risk of off-target genome edits (Bloomer et al., 2022; Kim et al., 2014). In non-recalcitrant crops, RNP complexes represent a highly promising alternative for genome editing via protoplast transformation. This strategy involves the direct delivery of the Cas9 protein complexed with sgRNA, forming a functional unit capable of inducing precise genomic modifications without the integration of transgenic sequences (Zhang et al., 2021). Model crops of agronomic interest such as potato (*Solanum tuberosum*) (González et al., 2020), tomato (*Solanum lycopersicum*) (Lin et al., 2022), tobacco (*Nicotiana tabacum*) (Liu et al., 2020), grape (*Vitis vinifera*) and apple (*Malus domestica*) cultivars (Malnoy et al., 2016) have been the subject of multiple investigations using this system, achieving the introduction of targeted mutations effectively and without leaving foreign genetic residues (Figure 3).

**FIGURE 4:**
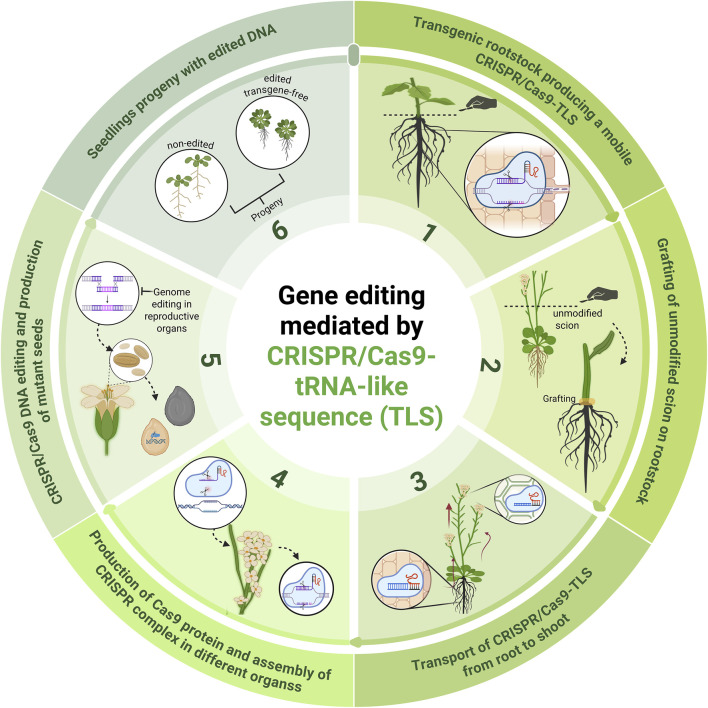
Plant genome editing using a CRISPR/Cas9-tRNA-like sequence (TLS). This strategy begins with a transgenic rootstock engineered to express a mobile CRISPR/Cas9-TLS construct (1). An unmodified (non-transgenic) scion is grafted onto this rootstock (2), enabling the long-distance transport of the CRISPR/Cas9-TLS from root to shoot (3). Once in the shoot, the system initiates production of Cas9 protein and assembly of CRISPR complex in different organs (4), enabling genome editing in reproductive tissues (5). This results in the generation of edited, transgene-free progeny (6), which segregate into mutant and wild-type lines. This approach offers a promising platform for transgene-free gene editing in crop breeding.

On the other hand, a major challenge in the gene editing of recalcitrant crops is their intrinsic difficulty for genetic transformation and *in vitro* regeneration, which limits the applicability of these technologies (Nivya and Shah, 2023). This limitation is particularly evident in many commercial cultivars, which often lack the capacity for efficient transformation. In addition, callus culture process is time-consuming and can lead to undesirable somaclonal variations, further complicating the development of stable edited lines (Wang and Wang, 2012). In this context, the incorporation of TLS motifs into the sgRNA of the CRISPR/Cas system is presented as an innovative strategy. These motifs allow the modified sgRNA to mobilize systemically through the phloem, reaching distant tissues that would otherwise be inaccessible for direct editing (Wu, 2023; Yang et al., 2023) (Figure 4). This strategy has been applied in model species such as *Arabidopsis thaliana* and *Brassica rapa*, by constructing 3′-end fusions of the Cas9 sequence gene and sgRNA sequences with TLS motifs, with the aim of facilitating the systemic transport of RNA from transgenic rootstocks to wild-type scions (Yang et al., 2023). This approach has made it possible to achieve heritable gene editing in recipient tissues without the integration of transgenes in the offspring, thus constituting a promising alternative for transgene-free gene editing in recalcitrant crops (Lyu, 2023).

We summarize that both strategies would be considered as possible ways to obtain transgene-free genome editing crops without the integration of exogenous DNA in the plant genome, and their applicability depends largely on the type of crops, the availability of efficient regeneration protocols and the technical conditions of the laboratory. CRISPR/Cas9-RNP would be useful for non-recalcitrant crops (p.e: potato, banana, strawberry, coffee) and CRISPR/Cas9-TLS offers an alternative and innovative pathway for recalcitrant crops (p.e: cacao), avoiding the need to directly edit protoplasts.

## 4 Potential applications in Peruvian agriculture

**FIGURE 5:**
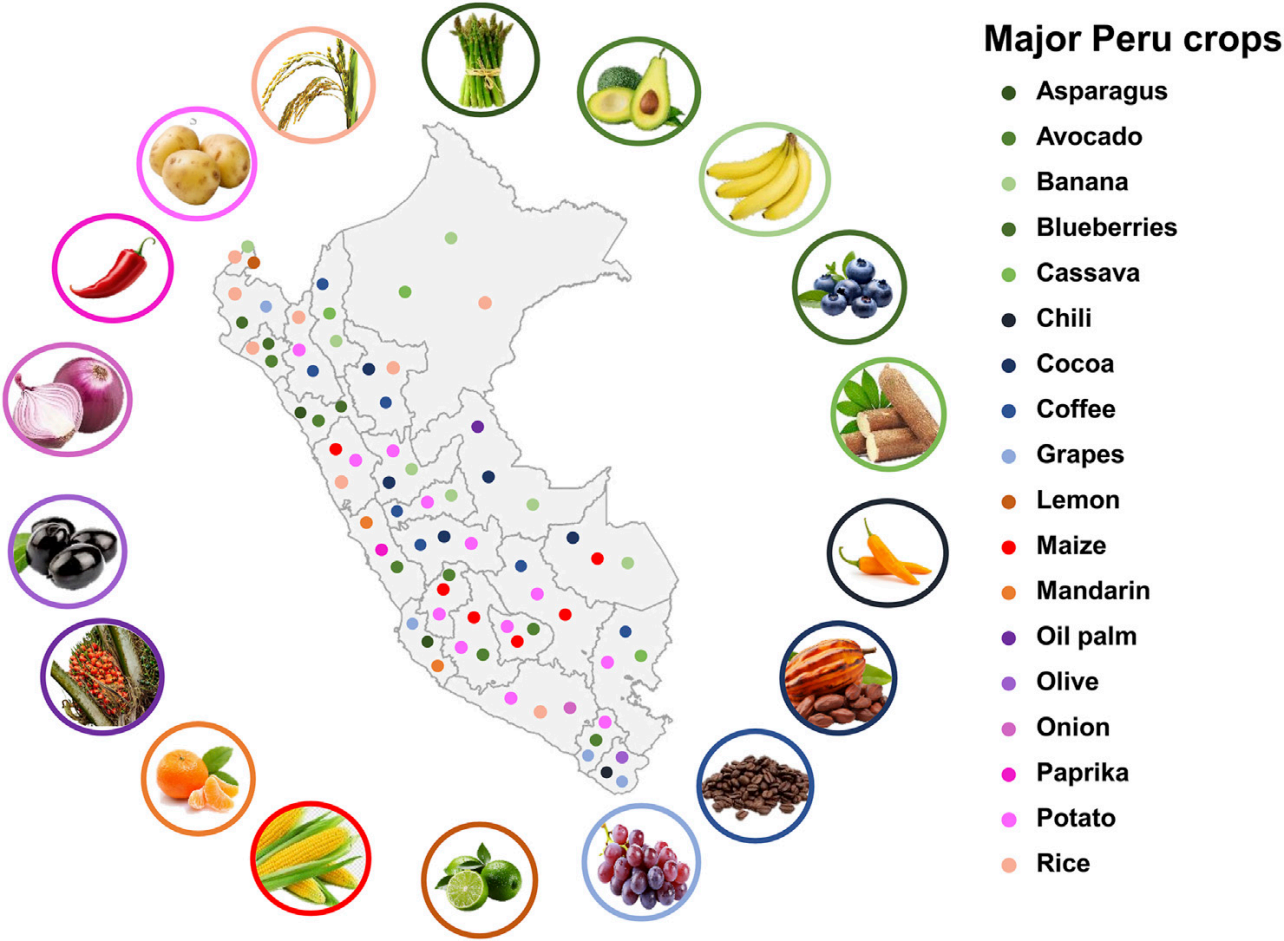
Map of Peru showing the main crops by regions with potential for gene editing research.

Plant gene editing could make a significant contribution to Peruvian agriculture by offering innovative solutions to enhance the productivity and sustainability of the agricultural sector (Zhang et al., 2018). These innovations could strengthen food security and the agricultural economy in Peru, especially in tropical, Andean and coastal regions, where the crops face production challenges in their specific agroecosystem (Reynel et al., 2013; USAID, 2017) (Figure 5). In this context, CRISPR/Cas and other gene editing technologies are important tools to drive sustainable and competitive agricultural development (Camerlengo et al., 2022).

One of the most significant advantages of gene editing is its potential to develop crops with enhanced resilience to climate change, a critical challenge for modern agriculture. Through targeted genetic and epigenetic modifications, it is possible to increase plant tolerance to adverse environmental conditions, such as drought and water scarcity (Shinwari et al., 2020). This is especially relevant for the Andean region, where drought is a constant threat to crops such as potato, maize, quinoa, beans and others crops. Recent studies have shown that CRISPR/Cas9 has the potential to modify important genes associated with drought resistance in maize (Liu et al., 2023). Shi et al. (2017) have shown that it is possible to adapt this crop to the dry environmental conditions of the country and thus increase its yields. Similarly, temperature-sensitive crops such as potatoes (Gangadhar et al., 2014), could benefit from genetic modifications designed to enhance their heat tolerance and safeguarding their yields in an environment of accelerated climate change (Chincinska et al., 2023).

In areas with frequent flooding, gene editing offers a promising solution by enabling the development of rice varieties with improved water-use efficiency. Recent studies have shown that it is feasible to increase the resistance of rice to both drought (Yang et al., 2022) and flooding by optimizing key physiological traits. These advancements include improved water retention under drought conditions and the ability to maintain gas exchange during submergence, thereby preventing cellular hypoxia and supporting plant growth in extreme environment (Yang et al., 2022).

Pest and disease management is another area where gene editing provides effective responses. Potato, a representative crop of Peru, has been investigated in order to enhance its resistance to pests such as potato tuber moth (Salim et al., 2024) and diseases such as late blight (*Phytophthora infestans*) (Karlsson et al., 2024; Zahid et al., 2024). The International Potato Center (CIP with headquarters in Peru), has been conducting research in Africa and Asia based on gene editing of this Andean tuber to generate more productive crops, resistant to diverse climatic conditions and capable of repelling pests or being immune to diseases (Sánchez-Valdivia, 2024). These advances, applied in Peru, would increase potato productivity and reduce pesticide use, thereby contributing to sustainable agriculture. In this context, we are conducting research using the RNP-mediated CRISPR/Cas9 system to inactivate polyphenol oxidases in native Peruvian purple potato cultivars and reduce enzymatic browning. Also, other crops, such as cotton and maize, have undergone genetic modifications to develop natural compounds that repel insects, reduce the need for insecticides and protect the environment (Meissle et al., 2022).

The use of improved crops, which leads to a reduction in the use of insecticides, not only benefits the environment, but also food safety. According to a report by the National Agricultural Health Service (SENASA) in 2022, analyses of food samples in Peru showed that the pesticide limits established in the Food Safety Law (Legislative Decree no. 1062) were exceeded in between 16% and 26% of samples. Tomatoes (77% of the samples), peppers and yellow peppers had concentrations above the permitted levels (Senasa, 2023). The results emphasize the importance of regulating the use of pesticides in Peruvian agriculture and promoting innovative plant biotechnology solutions to safeguard food safety and protect consumer health.

Improving the nutritional profile of crops is a critical objective in the context of Peruvian agriculture. Biofortification offers a viable approach to combat malnutrition in vulnerable rural populations by increasing the content and bioavailability of essential nutrients in staple foods (Goswami et al., 2022; Kiran et al., 2022). In 2023, Peru reported that 11.5% of children under 5 years of age were suffering from chronic malnutrition (Endes-Inei, 2024). This result is alarming, given that undernutrition during childhood can cause irreversible impacts on children’s cognitive and physical growth (Suryawan et al., 2022).

Plant breeding needs to be directed not only at increasing yield, but also incorporate strategies to enhance the nutritional quality of crops. Over the years, durum wheat varieties have achieved significant gain in productivity, but they exhibited a decline of 11%–25% in Fe^+2^ and Zn^+2^ concentrations, apparently due to a dilution effect (Murphy et al., 2008). Biofortification seeks to counteract this trend by simultaneously enhancing crop yields and increasing the concentration of essential micronutrients in staple cereals cultivated in Peru (Kadam et al., 2023). In addition, studies on crops such as potatoes, rice, cassava, tomatoes, maize, bananas, and carrots have shown that they have the potential to improve nutrient profiles and agricultural performance (Garg et al., 2018). However, recent studies emphasizes that the success of biofortification must be coupled with studies on the bioavailability of the nutrients to ensure that intended health benefits are realized (Huey et al., 2024).

Thus, gene editing plays a crucial role for advancement of agricultural innovation in agrifood systems to be become more sustainable, resilient and climate-adaptive, providing sufficient safe and nutritious foods for healthy diets under different agroecosystems of the countries, including Peru. However, a significant gap remains in the identification and functional characterization of new specific genes in crops that could be targeted through gene editing to achieved desirable agronomic and nutritional traits.

## 5 Regulatory policy in Peru and the moratorium on living modified organisms in the Latin American context

Most, if not all, countries that have ratified and implemented the Cartagena Protocol on Biosafety (CPB) will regulate transgenic organisms as living modified organisms (LMOs) based on their implementation of the CPB into national law. Peru introduced the LMO definition in its biosafety legislation in 1999 (Law no. 27104) and subsequently ratified in the CPB in 2004. However, with the appearance of targeted mutagenesis and other potentially non-transgenic techniques, various countries are adopting different regulatory approaches to the resulting products and this is a complicating factor not least in international trade with agricultural products. In Latin America, seven countries so far have introduced specific provisions addressing the regulatory status of the products of such precision breeding (Gatica-Arias, 2020; Rosado and Eriksson, 2022). Argentina was the pioneer in 2015 (Whelan and Lema, 2015) and since then also Brazil, Chile, Colombia and Paraguay have adopted similar approaches. This approach includes a pre-submission consultation where the regulatory status of the product is determined, most notably focusing on whether or not the final organism has a novel combination of genetic material. Guatemala and Honduras have adopted a bilateral agreement to facilitate commercial agricultural exchange between the two countries, including certain provisions on the regulatory status of the products of precision breeding. Peru has hitherto not adopted any official declaration beyond the adoption of the CPB, however initial discussions point to the possibility that some products derived from precision breeding may be treated as LMOs (Rosado and Eriksson, 2022). In this case, it remains to be seen what would be the definition of biotechnology-derived products that are not to be regulated as LMOs in Peru. It is important though that the lawmakers take into consideration the potential impact on international trade in the Latin American region, which will be hampered if countries adopt widely different approaches to the regulation of the products of precision breeding.

Peruvian regulations emphasize the protection of biodiversity and public health based on the precautionary principle. Although the objective of this strategy is to conserve natural resources, it has caused controversy by restricting the use of technologies that could help increase the sustainability and efficiency of agricultural production. The moratorium not only reflects concern about the potential socioeconomic and environmental consequences of LMOs, but also serves to protect traditional agricultural methods and ancestral practices.

However, transgene-free editing presents a promising approach to overcoming these limitations while ensuring environmental safety, given that the Peruvian government develops an enabling regulatory framework that does not put unnecessary restrictions on innovation. Given advancements such as RNP complexes or ‘mobile’ CRISPR/Cas9 — both transgene-free and widely applied in crops of agronomic importance—there is a need to reassess existing regulatory frameworks. Updating and approving these regulations would facilitate the safe and efficient adoption of biotechnological innovations in Peru, contributing to the sustainable development of agriculture.

In this context, cooperation between scientists, legislators, and farmers is crucial to creating an exemplary regulatory framework that promotes technological innovation while ensuring safety and environmental sustainability. Policies should consider all stakeholders’ needs and concerns and be guided by scientific knowledge. In addition, increasing public awareness of modern biotechnologies is essential and requires well-structured outreach and education programs.

## 6 Conclusion and future prospects

Gene editing, mediated by CRISPR/Cas system, particularly through transgene-free approaches in crops, offers promising opportunities for research, development, and practical application. This technology could significantly contribute to agricultural innovation within Peru’s agrifood systems by enhancing crop resilience and climate adaptability across the country’s diverse agroecosystem.

The adoption of transgene-free approaches, such as RNP-based editing and ‘mobile’ CRISPR/Cas9, could be a viable pathway to use biotechnological innovations in the Peruvian agriculture face to the strict provisions of the Peruvian moratorium on GMOs, ensuring greater compliance with biosafety standards.

Finally, the establishment of robust regulatory frameworks that balance technological innovation with ecological and social responsibility is crucial to fully harness the potential of gene editing and to advance a sustainable and globally competitive agricultural sector in Peru. Achieving this goal will require effective collaboration among scientists, policymakers, and farmers. Public awareness and education campaigns will also go a long way in promoting acceptance and understanding of this technology base on science.
